# Graded exercise with motion style acupuncture therapy for a patient with failed back surgery syndrome and major depressive disorder: a case report and literature review

**DOI:** 10.3389/fmed.2024.1376680

**Published:** 2024-04-08

**Authors:** Do-Young Kim, In-Hyuk Ha, Ju-Yeon Kim

**Affiliations:** ^1^Department of Acupuncture and Moxibustion, Jaseng Korean Medicine Hospital, Seoul, Republic of Korea; ^2^Jaseng Spine and Joint Research Institute, Jaseng Medical Foundation, Seoul, Republic of Korea

**Keywords:** failed back surgery syndrome, major depressive disorder, rehabilitation therapy, motion-style acupuncture therapy, graded exercise therapy

## Abstract

Effective treatment of failed back surgery syndrome (FBSS) remains challenging despite urgent medical attention requirements. Depression is a contributing factor to the development and poor prognosis of FBSS, and vice versa. We report the case of a patient with FBSS and major depressive disorder (MDD) treated with graded exercise combined with motion-style acupuncture therapy (MSAT). A 53-year-old male veteran who had undergone lumbar discectomy and laminectomy with instrumented fusion was admitted to the hospital with re-current back pain and radiative pain in the left leg. The effects of failed surgery triggered MDD as a comorbidity. After a six-week routine treatment without remarkable improvement, a three-week program of graded exercise with MSAT was applied. The numeric rating scale (NRS) and short form-36 (SF-36) were used to assess low back pain with radiating leg pain, and daily functioning levels, respectively. The voluntary walking distance of the patients was measured. To analyze the therapeutic effects and other applications of the intervention, we surveyed clinical trials using MSAT or graded exercise therapy (GET). Three weeks of graded exercise with MSAT reduced physical and mental functional disabilities (SF-36, physical component: 15.0 to 37.2, mental component: 21.9 to 30.1) as well as the intensity of low back pain and/or radiative leg pain (NRS: 50 to 30). Furthermore, as the therapeutic intensity gradually increased, there was a significant corresponding increase in daily walking distance (mean daily walking distance, the first week vs. baseline, second, and third week, 3.05 ± 0.56: 2.07 ± 0.79, 4.27 ± 0.96, and 4.72 ± 1.04 km, *p* = 0.04, *p* = 0.02, and *p* = 0.003, respectively). Three randomized controlled trials of GET were included, all showing statistically significant antidepressant effects in the diseased population. Graded exercise with MSAT may be an effective rehabilitative therapy for patients with FBSS and MDD who have impaired daily routines.

## 1 Introduction

Failed back surgery syndrome (FBSS) is a post-spinal surgery condition characterized by persistent or recurrent spinal pain and/or radiating pain in the lower extremities (1). The incidence of FBSS is estimated to range from 10 to 40% after lumbar laminectomy, with or without instrumented fusion (2). Recently, owing to the increased demand for lumbar surgeries to treat degenerative spinal diseases, that is, an approximately 140% increase from 2004 to 2015 in the United States and 2-fold increase from 1997 to 2018 in Finland, FBSS requires medical attention (3, 4). Patients with FBSS exhibit debilitated mental and physical functions and experience a lower health-related quality of life (QoL) than patients with cancer or stroke (5). The estimated annual cost of medical and productivity losses ranges from US $22,403 to US $26,170 per patient in Washington State (6).

Factors contributing to the development of FBSS include recurrent spinal disease, postoperative infection, nerve injury, and psychological disorders such as depression (1). Psychiatric problems are frequently encountered in cases of medically unexplainable pain with a challenging pathophysiology. Patients with FBSS have a significantly higher risk of developing mental disorders (7). According to the National Inpatient Sample database, the incidence of comorbid depression in hospitalized patients with FBSS was estimated to be 23% in 2015, and this was dominant in the working-age population (8). Moreover, chronic pain and mood disorders mutually contribute to a poor prognosis (9). Given the absence of an established treatment for FBSS accompanied by the unexplainable pain, not even with depression, the development of valid therapeutic approaches for FBSS with depressive disorder is imperatively required.

Acupuncture and exercise therapy are commonly recommended as effective non-pharmacological treatments for chronic low back pain (LBP) (10). The mechanism of pain relief provided by acupuncture involves neuronal modulation within the central nervous system (CNS) through peripheral stimuli (11). A modification of acupuncture, motion-style acupuncture therapy (MSAT), wherein acupuncture is combined with coordinated motion, demonstrated a decent analgesic effect on chronic LBP in spinal degenerative disorders such as herniated intervertebral disc (HIVD) (12, 13). MSAT reinforces the stimuli to invade tissues using the targeted muscles. In contrast, graded exercise therapy (GET) is a type of physical training in which the intensity of exercise is gradually increased (14). GET strengthens physical capacity and provides psychological encouragement by demonstrating objective improvements in patient performance (15). GET has been shown to be effective in ameliorating the conditions of patients with cancer with comorbid mental disabilities such as depression, anxious mood, and catastrophizing (16). The respective therapeutic effects of MSAT and exercise for LBP or depressive mood are widely recognized; however, the clinical application and synergies of combining these treatments are novel for pain or mental disorders. Considering the pain-relief effect and neuronal activity improvement provided by acupuncture, we hypothesized that MSAT with simultaneous GET would improve both physical and mental health in chronic pain with depressive disorder.

Herein, we evaluated the effect of GET with MSAT in a patient with FBSS and major depressive disorder (MDD). We also undertook a literature review of clinical trials that used MSAT or GET for depressive mood therapy in a diseased population to corroborate the findings of the case study and further clinical applicability.

## 2 Case presentation

### 2.1 Patient characteristics and medical history

A 53-year-old Asian male veteran was admitted to Jaseng Hospital of Korean Medicine on October 7, 2022, presenting complaints of LBP and numbness in the left shin and hallux, which initially manifested 3 years prior. His anthropometric measurements include a height of 168.3 cm, weight of 65.2 kg, and a body mass index (BMI) of 23.0, positioning him on the border between normal and overweight classifications. Over the past 3 years since the onset of pain, he has maintained a sedentary lifestyle, engaging in less than 30 min of walking per day. He adheres to non-smoking and non-drinking habits and reports no comorbidities or significant familial history of underlying diseases. The range of motion (ROM) of the lumbar spine was limited only in flexion/extension, measuring 70/10 degrees respectively, and a negative straight leg raise test was observed. The blood and urine analyses conducted upon admission, which encompassed a complete blood count, glucose/lipid metabolic panel, liver and kidney function tests, and inflammatory markers, did not reveal any remarkable findings.

**Figure 1:**
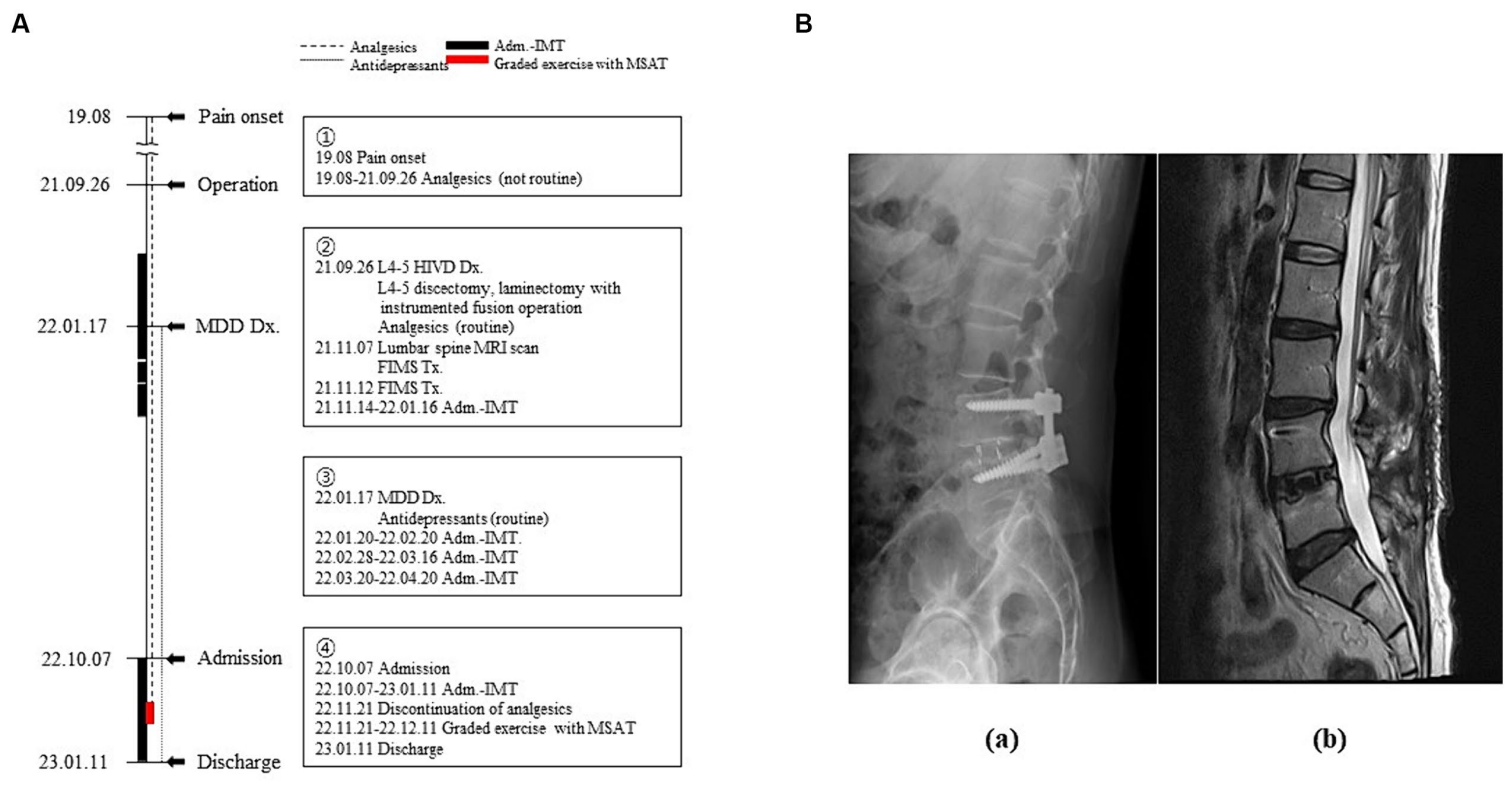
Timeline of the patient’s medical history. **(A)** Timeline of the medical events between the onset of the pain and the current treatment period. Narrative demonstrations of the timeline are in the boxes. **(B)** Radiological images of the lumbar spine (a: lateral view of X-ray image, b: Sagittal section of T2-weighted magnetic resonance image). Adm, admission; Dx, diagnosis; FIMS, functional intramuscular stimulation; HIVD, herniated intervertebral disc; IMT, integrative medicine treatment; MDD, major depressive disorder; MRI, magnetic resonance imaging; MSAT, motions style acupuncture therapy; Tx, treatment.

In his medical history, the patient had been diagnosed with L4–5 HIVD and had undergone discectomy and laminectomy with instrumented fusion in September 2021. Before diagnosis and surgery, he received no treatment other than arbitrary analgesic administration when the pain was severe. Although the surgery was successful with no abnormal findings on magnetic resonance imaging (MRI) or computed tomography (CT), the patient had no symptomatic changes in the lower back or lower extremities (Figure 1B). Subsequently, he had to be discharged from the army because of disturbance in his activities of daily living, which had become strikingly worse than before becoming diseased. Moreover, following two ineffective functional intramuscular stimulation (FIMS) treatments, the patient developed helplessness and hopelessness. This triggered depression, manifesting as sleep disturbance, reduced appetite, pessimism regarding his symptoms, and a subsequent attempted suicide. Later that year, he was diagnosed with MDD. Four admissions for integrative medicine treatment, before and after the diagnosis of MDD, did not indicate any pain management strategies (Figure 1A).

### 2.2 Diagnosis and treatment

The diagnosis of FBSS was determined based on the persistent LBP with left radiative pain and numbness that existed despite surgery being performed in 2021. Furthermore, MDD was diagnosed in accordance with the Diagnostic and Statistical Manual of mental disorders-5 (17).

Routine treatments, as standardized by practice guideline (18), included acupuncture with electronic stimulus, pharmacopuncture, herbal medicine, analgesics, and psychopharmacotherapy which were prescribed and administered, since his admission in October 2022, by the psychiatric clinics that had confirmed the diagnosis of MDD (Supplementary Table 1). Since the 6-week routine treatment did not provide remarkable improvement, graded exercise with MSAT was additionally performed after excluding analgesics from the routine treatment. It was prospectively employed with the expectation of curative effects on debilitating pain and depressive mood by referring to its successful application in patients with myalgic encephalomyelitis/chronic fatigue syndrome (ME/CFS) (19).

MSAT is a physical restoration technique performing the specific movements with needles inserted into the muscles associated with restricted motion (12). The treatment method used in this case combines the characteristics of MSAT, usually employed as a one-off treatment, with GET, which gradually increases the distance and weight. The acupoints utilized were both sides of LR3, ST36, and BL25. These points were chosen to stimulate muscles associated with walking pain (BL25: erector spinae, ST36: tibialis anterior) as well as brain activities related to pain perception and emotional processing (LR3) (20). Needles measuring 0.25 × 30 (DongBang Co., Seoul, Korea) were used. With the needles retained at the acupoints, the patient was instructed to walk at a pace of one step per second, while carrying 0–2 sandbags, each weighing 800 g. (21). Exercise was performed on weekdays for 3 weeks. The number of exercise sets (50 m per set) and sandbags were gradually increased based on the patient’s exercise performance under the physician’s judgment. The rest intervals between sets are 30 s.

### 2.3 Course of symptom and physical performance

The numeric rating scale (NRS) for LBP and radiating leg pain was recorded at baseline and every end of weekly treatments. Also, daily functioning levels was assessed with short form-36 (SF-36) at baseline and the end of the intervention (22). In addition, spontaneous daily walking distances, except for intervention-associated steps, were measured using a Samsung Health Pedometer (Samsung Electronics Co., Suwon, Korea).

**Figure 2:**
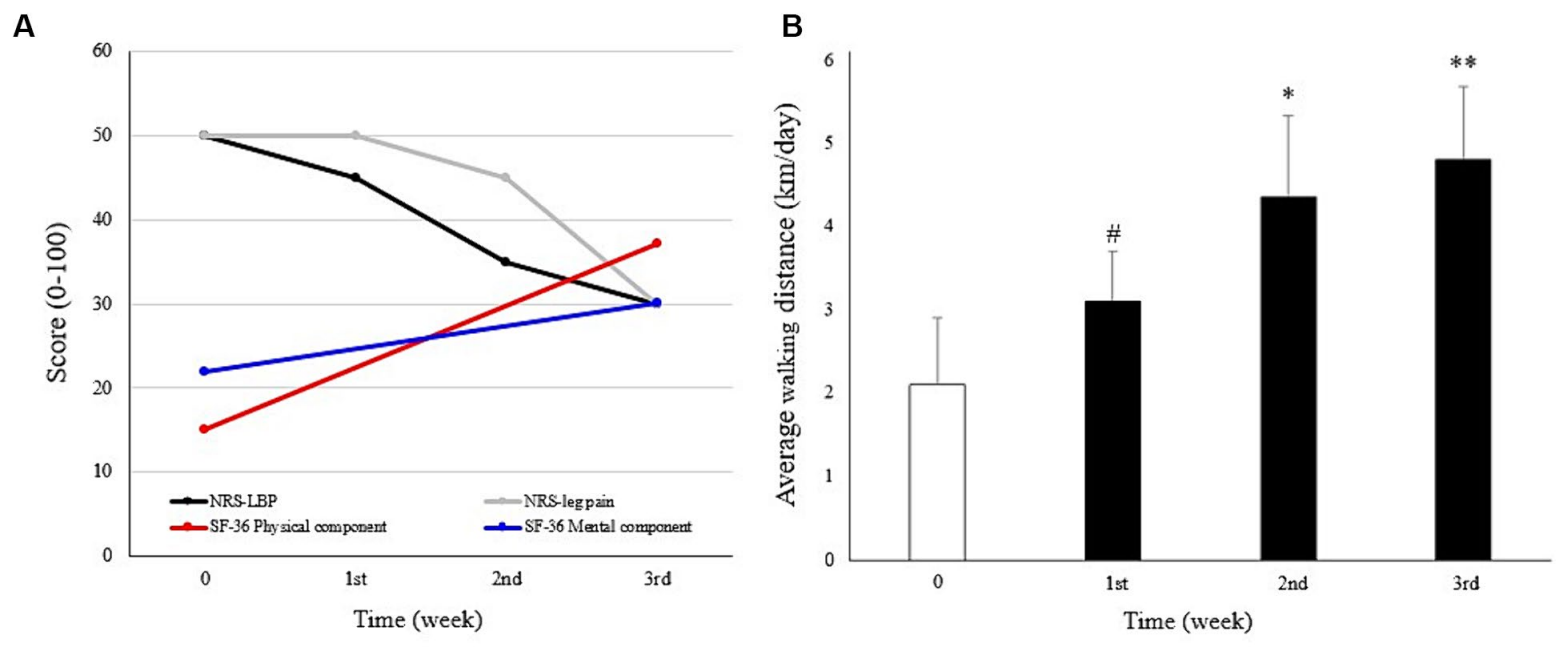
Course of symptoms. **(A)** Changes in low back pain (LBP), leg pain, physical and mental health scores, measured using the numeric rating scale (NRS) and Short Form-36 (SF-36), respectively. **(B)** Mean daily walking distances were calculated at baseline (Week 0, before the intervention) and the first to third week during which the intervention was applied, with gradually increased exercise intensity. #*p* < 0.05, compared with baseline. *p < 0.05; **p < 0.01 compared with the first week. Mann–Whitney U test was used to analyze statistical significance.

As shown in Figure 2A, 3 weeks of graded exercise with MSAT reduced the intensity of LBP and radiative leg pain (NRS: 50 to 30), as well as improved both physical and mental health (SF-36, physical component: 15.0 to 37.2; mental component: 21.9 to 30.1). Furthermore, the physical function of the SF-36, which includes general activities of daily living including walking, climbing stairs, and carrying objects, showed improvement. However, his emotional state, particularly in feeling blue, nervous, and worn out, still persisted, as evidenced by the SF-36 mental component scores.

The intensity of the intervention was gradually escalated. During the initial week of treatment, the patient executed two sets (100 m) daily without the use of sandbags. Subsequently, in the second week, the regimen progressed to three sets with the addition of one sandbag (800 g), and by the third week, the patient advanced to four sets while incorporating two sandbags.

Similarly, the vitality and physical function of the patient improved as daily walking distance increased. An immediate increase in the distance was shown in the first week (3.05 ± 0.56 km compared to that of the previous week at 2.07 ± 0.79 km, *p* = 0.04). This improving pattern was maintained in the following 2 weeks as well (4.27 ± 0.96 and 4.72 ± 1.04 km compared to the distance in the first week, *p* = 0.02 and *p* = 0.003, respectively) in accordance with gradually intensified exercise (Figure 2B). No adverse events were observed during the treatment.

### 2.4 Literature review

A literature survey was conducted using a single database: PubMed, up to December 2024, to identify clinical trials that employed MSAT or GET to treat depressive mood as a symptom in diseased populations. The inclusion criteria were as follows: (1) studies following a randomized controlled trial (RCT) design and assessed the effects of MSAT or GET, (2) studies being conducted on participants who were diseased adults, and (3) studies that evaluated changes in depressive mood using a relevant measuring tool. The search term used was “(motion style acupuncture or graded exercise therapy) and (depression) and (randomized controlled trial),” and references were screened by examining their titles, abstracts, and full texts.

Out of the total of 123 surveyed initial articles, none included RCTs assessing the MSAT for its antidepressant effect. Consequently, three RCTs with GET were reviewed, and the characteristics of the trials are summarized in Table 1. Two RCTs were conducted on patients with ME/CFS and presented statistically significant improvements in depressive mood and fatigue following therapy (23, 25). The antidepressant effect of GET was also demonstrated in another RCT that included patients with chronic neck pain (24).

**Table 1:** Characteristics of the studies in the literature review.

Disease [reference]	No. of participants (Female, Mean age)	Intervention (Control)	Measurement ^A^	Statistical significance	Adverse event
ME/CFS (23)	211 (167, 38.4)	Guided, graded exercise self-help with usual care (Usual care)	Chalder fatigue scale SF-36 physical function HADS	*P* < 0.01	None
Chronic neck pain (24)	200 (149, 45.1)	GET with pain education (Pain education)	SF-36 NDI BDI	Physical function: *P* < 0.05 Mental function: *p* = 0.01 NDI: Not significant BDI: *p* = 0.01	One event of increasing pain during exercise
ME/CFS (25)	61 (47, N.A)	Graded exercise with pacing (Relaxation)	Chalder fatigue scale HADS	Mental fatigue: *p* < 0.05 Physical fatigue: N.S HADS: *p* < 0.05	N.A

^A^Presents measurements employed for assessing chief complaint-related symptoms of each disease and depressive mood. BDI, Beck Depression Inventory; GET, graded exercise therapy; HADS, Hospital Anxiety and Depression Scale; ME/CFS, myalgic encephalomyelitis/chronic fatigue syndrome; NDI, Neck Disability Index; N.A, not available; N.S, not significant; SF-36, 36-item Short Form health survey.

## 3 Discussion

Pain commonly accompanies depressive symptoms (26). Greater pain severity and refractory treatment outcomes are associated with more depressive symptoms and worse depression outcomes (27). In our case, the patient had a negative and hopeless mood, exacerbated by impairments on his daily living, such as restricted mobility, driven by ineffective therapeutic interventions. This involved the conservative and symptomatic management for medically unexplainable pain he had been undergoing without abnormal findings on MRI or CT. Accumulating evidence suggests that spinal cord stimulation can reduce pain and improve physical function in patients with FBSS but would not affect mental health (28). Despite 1.5 months of routine care, including paravertebral electroacupuncture, depressive symptoms and pain persisted in our patient (Supplementary Table S1). However, we demonstrated how we used GET and MSAT to improve pain outcomes as well as physical and mental function in a patient with FBSS and MDD.

Acupuncture is recommended as an effective nonpharmacological intervention for the treatment of chronic LBP (10). Needling of abnormally strained muscles, known as trigger points, can ameliorate spasticity and pain in patients with LBP (29). MSAT reinforces the stimulus quantity of acupuncture by moving the needled muscles. It promotes the pain-relief effect of therapy such that the patient gradually expands their limited ROM, leading to restored physical function (13). The analgesic effect of MSAT in this case, which is not achieved through conventional acupuncture, may arise from the release of instrumented surgery-derived hyper-strained tissues by activating muscles involved in gait cycles. The selected acupoints, BL25 and ST36, were strategically chosen due to their proximity to primary pain regions and their involvement in locomotion. Additionally, the pain regulation effect of acupuncture includes the modulation of neuronal activity in the CNS (11). The peripheral stimulus-derived opioids 5-hydroxytryptamine (5-HT) and norepinephrine in the CNS are believed to regulate inflammatory and neuropathic pain (30, 31). A study using electroencephalography suggested that pain improvement following electrocutaneous treatment in patients with FBSS was associated with increased cerebral activity, primarily in the anterior cingulate gyrus, which participates in pain and emotion processing (32, 33). Likewise, the acupoint LR3 employed in this case is recognized for its modulatory effects on pain perception and emotional functions (20). By increasing the peripheral stimulus, MSAT may provide a greater analgesic effect with neuronal modulation in the CNS than conventional acupuncture therapy (Figure 2A).

Exercise has been used in musculoskeletal reinforcement and psychological therapy (15). Regarding chronic pain, exercise therapy is a nonpharmacological and noninvasive approach recommended by the Centers for Disease Control and Prevention (34). Activation of the deep trunk muscles by exercise and performing of complex functional tasks is recommended as an effective noninvasive treatment for chronic LBP (35). In our patient, exercise with MSAT focused on the trunk muscles via acupuncture and slow walking while maintaining the body’s balance. Gradually increasing the intensity and repeating the processes provided cardiovascular training and muscle strengthening. In addition, aerobic exercise has been shown to have a positive mood regulation effect in patients with depression (36). Cardiovascular exercise is believed to promote 5-HT metabolism in the CNS and reconfigure the brain structure in patients with depression, thereby improving neuroprocessing and delaying cognitive degradation (37). Therefore, the graded exercise technique used may have contributed to mood regulation and pain control in our case (Figure 2A).

During the exercise, the physician closely guided and corrected the posture of the patient in a step-by-step manner and encouraged him to perform more tasks by demonstrating the results of what had been achieved objectively. Thus, the physician provided not only exercise feedback but also mental support. Cognitive behavior therapy, a type of mental support intervention, has been applied to chronic pain, including LBP, in which aberrant pain perception caused by heightened interoceptive awareness is a common clinical feature (38, 39). Likewise, the GET also has psychological benefits as it provides patients with confidence through a gradual increase in physical performance. In this context, cognitive behavior therapy and GET are recommended interventions for debilitating mental symptoms such as those of in ME/CFS (19). Therefore, it can be suggested that the mental-supportive features of graded exercise with MSAT might have motivated our patient to increase spontaneous activities (Figure 2B).

We prospectively adopted MSAT combined with GET as a therapeutic approach for a patient with FBSS and MDD, expecting amelioration of the low mood and pain. As a positive outcome was observed in this case, we analyzed its clinical utility by surveying RCTs to evaluate the antidepressant effects of MSAT or GET in diseased participants. All three RCTs we reviewed employed GET; the results demonstrated statistically significant improvements in the patients’ depression scores on the Beck Depression Inventory (BDI) or Hospital Anxiety and Depression Scale (HADS), compared with the scores of those in the control group (Table 1). Regarding the two RCTs for ME/CFS, unlike the overall positive outcomes of mental function scores in both trials, the results for physical fatigue were not aligned in the same positive direction (23, 25). Similarly, psychological impairment was significantly improved by GET in another RCT among patients with chronic neck pain, whereas the physical function in the neck measured by the Neck Disability Index was not (24). This might be due to the immoderate application on the patient vulnerable for excision, and one adverse event of increasing pain was reported. In fact, one survey on patients with ME/CFS reported that approximately 79% of respondents experienced their health worsening due to the GET (40). This implies that the clinical adoption of the GET requires the intensity of tasks to be adjusted considering the patient’s physical capacity and the possibility of increased pain. Regarding FBSS, moderate exercise enhances daily living, strength, and fearlessness in patients (41). Therefore, we hypothesized that MSAT assisted in providing adequate exercise intensity and pain control in our patient. The results of the case study and literature review indicate that graded exercise with MSAT may offer effective rehabilitation for individuals suffering from depression induced by pain disorders, and vice versa, facilitating a resumption of their daily activities.

This case study has several limitations. First, follow-up data were not available after the patient was discharged from the hospital, which can induce uncertainty regarding therapeutic effects and its generalizability, such as the Hawthorne effect. Second, routine integrative Korean medicine treatments were used previously and simultaneously with the intervention, which could have induced synergistic therapeutic effects. Third, depression-specific scales such as the BDI or the HADS were not used for evaluating the depressive status of the patient. While our primary focus lay in functional rehabilitation utilizing the SF-36, employing measurements specialized for MDD could provide more explicit assessments of mental status. The SF-36 survey is originally intended for measuring the QoL of patients, and we employed it considering the etiological history of this case, where restrictions in daily living, closely related to QoL, resulting from failed surgery caused depression. Nonetheless, to our knowledge, this is the first report of successful treatment of FBSS and MDD, unresponsive to usual medications, by employing graded exercise with MSAT. Further, there were no adverse events noted in the patient. However, to facilitate the clinical use of this intervention, further well-designed clinical trials on its efficacy and safety are warranted.

## Data availability statement

The original data presented in the study are included in the article/Supplementary material, further inquiries can be directed to the corresponding author.

## Ethics statement

The ethical review and approval of this study were waived by the Institutional Review Board of Jaseng Hospital of Korean Medicine, Seoul, Korea (IRB file No. JASENG 2023–12-002) for the study design: a single case study with literature review. The studies were conducted in accordance with the local legislation and institutional requirements. The participants provided their written informed consent to participate in this study. Written informed consent was obtained from the minor(s)’ legal guardian/next of kin for the publication of any potentially identifiable images or data included in this article.

## Author contributions

D-YK: Conceptualization, Investigation, Methodology, Visualization, Writing – original draft, Writing – review & editing. I-HH: Conceptualization, Writing – review & editing. J-YK: Supervision, Visualization, Writing – review & editing.

## Figures and Tables

**Table:** 

BDI	Beck depression inventory
CNS	Central nervous system
CT	Computed tomography
FBSS	Failed back surgery syndrome
GET	Graded exercise therapy
HADS	Hospital anxiety and depression scale
HIVD	Herniated intervertebral disc
LBP	Low back pain
MDD	Major depressive disorder
ME/CFS	Myalgic encephalomyelitis/chronic fatigue syndrome
MRI	Magnetic resonance imaging
MSAT	Motion-style acupuncture therapy
NRS	Numeric rating scale
RCT	Randomized controlled trial
ROM	Range of motion
SF-36	Short form 36
