# Supplementary material for: Graded exercise with motion style acupuncture therapy for a patient with failed back surgery syndrome and major depressive disorder: a case report and literature review
Source: Front Med (Lausanne). 2024 Apr 8;11:1376680. doi: 10.3389/fmed.2024.1376680 (PMC11034520; doi:10.3389/fmed.2024.1376680)
Supplement: Supplementary file 1 [file Table_1.docx]

Supplementary Material

**Supplementary Table 1.** Routine treatment

|  | |
| --- | --- |
| **Items** | **Treatment details** |
| Acupuncture | Acupuncture therapy with simultaneous electronic stimulus and intramuscular Shinbaro 2^A^ injection were conducted twice daily on ashi-points in the low back and left shin using infrared rays |
| Electroacupuncture |  |
| Pharmacopunture |  |
| Herbal medicine | |
| Yonggakyodaeyoung-tang | *Cervus elaphus*, *Lycium chinense,* *Angelica gigas*, *Eucommia ulmoides*, *Acyranthes bidentata*, *Glycyrrhiza uralensis*, *Geoclemys reevesii*, *Lashiosphaera nipponica,* *Cinnamomum cassia*, *Carthamus tinctorius*, *Rehmanniae radix* Preparata |
| Chungshinbaro-pill | *Poria cocos*, *Panax ginseng*, *Acyranthes bidentata*, *Asini qelatinum,* *Rehmannia glutinosa*, *Cervus elaphus*, *Apis cerana,* Cibotii Rhizoma, *Eucommia ulmoides,* *Acyranthes bidentata*, *Ledebouriella seseloides*, *Eleutherococcus sessiliflorus*, *Scolopendra subspinipes,* *Atractylodes japonica* |
| Woohwanggamek-pill | *Dioscorea polystachya*, *Glycyrrhiza uralensis,* *Panax ginseng,* *Typha orientalis*, Massa Medicata, Bubalus Bubalis, Glycine Semen, *Cinnamomum cassia*, *Asini qelatinum,* *Paeonia japonica*, *Liriope platyphylla,* *Scutellaria baicalensis*, *Angelica gigas*, *Ledebouriella seseloides*, *Atractylodes japonica*, *Bupleurum falcatum*, *Latycodon grandiflorum,* *Zizyphus jujuba,* *Triticum aestivum*, *Aquilaria agallocha, Dryobalanops aromatica* |
| Analgesic | |
| Aceclofenac | 200.0 mg 2T #2 |
| Acetaminophen | 325.0 mg 2T #2 |
| Tramadol Hydrochloride | 37.5 mg 2T #2 |
| Afloqualone | 40.0 mg 2T #2 |
| Tramadol Hydrochloride (injection) | 50.0 mg 1A #1 |
| Antidepressant | |
| Lorazepam | 0.5 mg 1T #1 |
| Quetiapine | 25.0 mg 2T #1 |
| Aripiprazole | 2.0 mg 1T #1 |
| Duloxetine | 60.0 mg 1C #1 |
| Mirtazapine | 15.0 mg 1T #1 |
| Trazodone | 25.0 mg 1T #1 |
| ^A^ Kim et al. Effects of the administration of Shinbaro 2 in a rat lumbar disk herniation model. Frontiers in Neurology. 2023;14:1044724. | |
